# CDCA5 promoted cell invasion and migration by activating TGF-β1 pathway in human ovarian cancer cells

**DOI:** 10.1186/s13048-024-01393-5

**Published:** 2024-03-27

**Authors:** Qingsong Zhang, Rong Zhang, Yuzhi Li, Xiaojun Yang

**Affiliations:** 1 Department of Obstetrics and Gynecology, The First Affiliated Hospital of Soochow University, 188 Shizi Road, Suzhou, 215006 Jiangsu People’s Republic of China; 2 Department of Gynecological Oncology, The First Affiliated Hospital of Bengbu Medical University, Bengbu, 233004 Anhui China

**Keywords:** CDCA5, TGF-β1, Ovarian cancer, Proliferation, Metastasis

## Abstract

**Background:**

The gene cell division cycle associated 5 (CDCA5), also called sororin, has oncogenic characteristics and is upregulated in various carcinomas. Nevertheless, the involvement of CDCA5 in ovarian cancer (OC), a highly aggressive form of cancer, and the underlying mechanism of metastasis remain inadequately investigated.

**Results:**

The bioinformatics data revealed a negative correlation between the patient’s survival and CDCA5 expression, which was overexpressed in OC. Functional assays also confirmed high expression levels of CDCA5 in OC tissues and cells. This suggests that CDCA5 may potentially enhance the motility, migration, and proliferation of OC cells invitro. It impedes DNA damage and apoptosis in OC cells, inhibiting xenograft development in nude mice. The RNA sequencing results suggest CDCA5 is majorly associated with biological functions related to the extracellular matrix (ECM) and influences the transforming growth factor (TGF) signaling pathway. Moreover, subsequent functional investigations elucidated that CDCA5 facilitated the migration and invasion of OC cells viathe TGF-β1/Smad2/3 signaling pathway activation.

**Conclusions:**

CDCA5 may be a strong potential therapeutic target for the treatment and management of OC.

**Supplementary Information:**

The online version contains supplementary material available at 10.1186/s13048-024-01393-5.

## Introduction

The fifth-largest cause of cancer-related mortality in women is ovarian cancer (OC) [1], with less than 50% of patients surviving five years after diagnosis [2]. A considerable number of patients diagnosed with OC exhibit the presence of metastases in various organs at the time of their initial presentation. This situation poses a challenge in attaining complete tumor removal with cytoreductive surgery, resulting in an unfavorable prognosis of the disease [3]. Hence, it is crucial to understand and elucidate the molecular pathways implicated in the progression and metastasis of OC to target it for therapeutic interventions effectively.

The TGF-β signaling system has significant importance in metazoan biology, serving as a pivotal component. Dysregulation of this pathway has been implicated in the formation of tumors. This cytokine family has several members that occur in various forms, such as TGF-β1, -β2,and -β3. Among these, TGF-β1 is the most often expressed and extensively investigated isoform [4]. Once activated, TGF-β transmits information through the process of phosphorylation of Smad transcription factors, notably Smad2 and Smad3. Recent research indicates that TGF-β can orchestrate fibrosis and facilitate epithelial-mesenchymal transition (EMT) [5], hence contributing to the development of fibrotic disorders [6] and the spread of tumors [7].

Cell division cycle associated 5 (CDCA5) was initially identified during the screening process for genes related to cell cycle-related transcripts [8]. CDCA5 gene encodes a protein, also known as sororin, which is crucial for attaching and segregating sister chromatids during mitosis [9]. It also facilitates the repair of DNA and maintenance of cohesion on chromosomes during meiosis in mammalian oocytes [10]. Recent investigations have shown the significant involvement of CDCA5 in facilitating the proliferation of several cancer types, including breast cancer [11], liver cancer [12], and prostate cancer [13]. According to research using integrated bioinformatics methods, CDCA5 is linked to a poor prognosis in OC [14]. Nevertheless, the extent to which CDCA5 contributes to OC needs to beinvestigated.

The present study evaluated the biological roles of CDCA5 in OC using in vivoand in vitro experimentation. The molecular mechanism underlying the function of CDCA5 inpromoting OC metastasis was also explored. Specifically, the involvement of the TGF-β pathway, which is known to be enriched by transcriptome alterations, was investigated. The current research will contribute to a greater comprehension of CDCA5 and provide novel insights for OC treatment strategies.

## Results

### Abnormal

Expression of CDCA5 inOvarian Cancerand its Prognostic Significance.

**Fig. 1:**
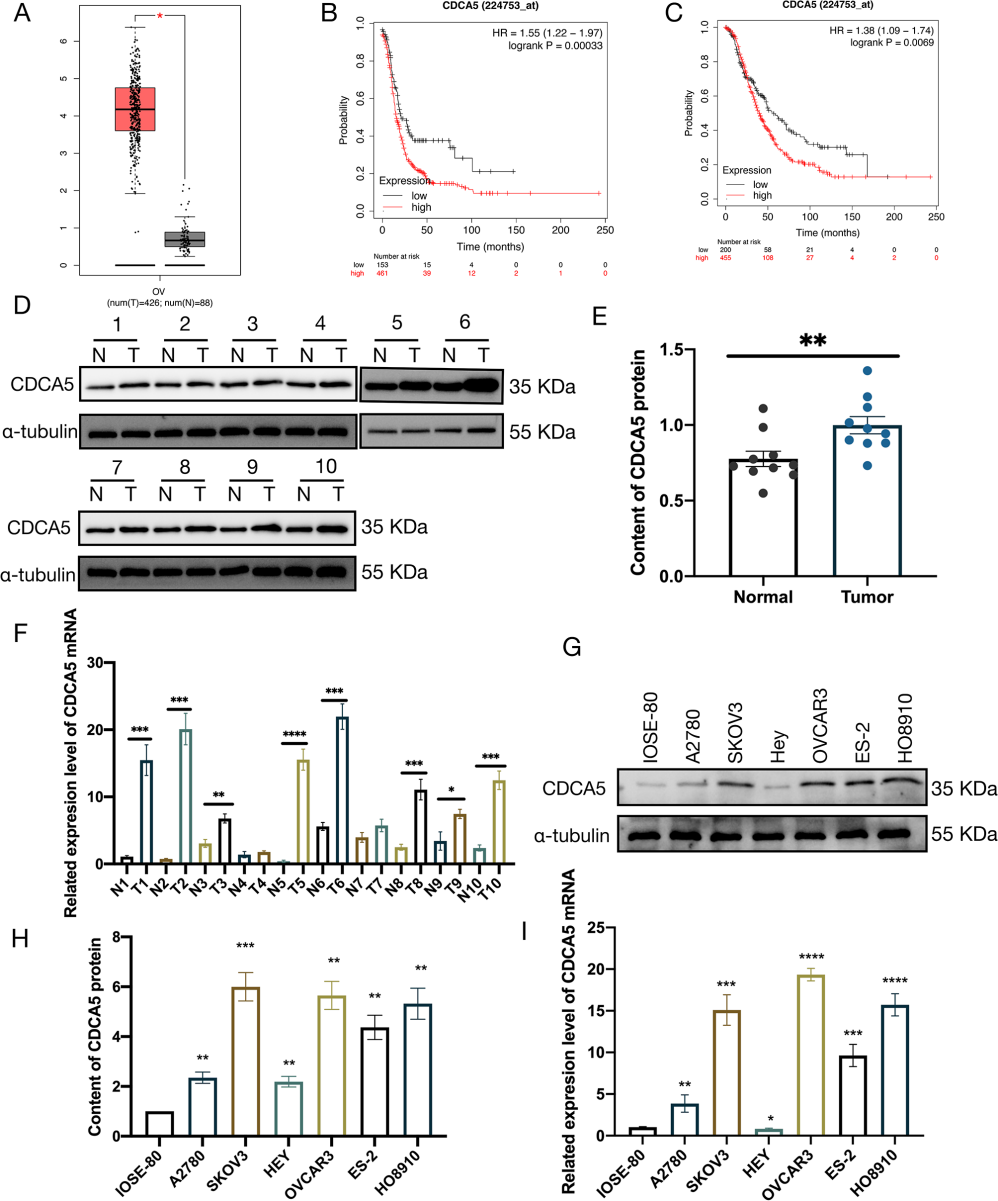
Overexpression of CDCA5 showed a positive correlation with poor prognosis in OC. **A** The GEPIA website generates a boxplot of CDCA5 expression in both normal and tumor ovarian tissues. **B**The KM Plotter database made KM curves of associations between CDCA5 expression levels and PFS in OC patients. **C** The KM Plotter database was also used to generate KM curves displaying the relation between CDCA5 expression levels and OS in OC patients. **D, E** The WB analysis of CDCA5 protein in normal (N) and OC tumor (T) tissues. **F** The CDCA5mRNA levels in normal(N) and OC tumor (T) tissues were examined by qRT-PCR. **G, H** The expression of CDCA5 in IOSE-80 cells and six human OC cell lines were analyzed by WB. **I** The qRT-PCR evaluation of CDCA5 mRNA levels in IOSE-80 cells and six human OC cell lines

In order to investigate the significance of the CDCA5 genein OC, initially, the expression levels of CDCA5 in malignant tumors were examined using the Gene Expression Profiling Interactive Analysis (GEPIA) website. The findings revealed a substantial upregulation of CDCA5 mRNA expression in OC (Fig. 1A). The Kaplan-Meier curves demonstrated that patients with OC exhibited an elevated level of CDCA5 gene expression with a lower progression-free survival (PFS) and overall survival (OS) compared to those with a low level of CDCA5 expression (Fig. 1B and C). Based on the obtained bioinformatics findings, it wasevident that CDCA5 had a potential significance that requires additional research, especially in the context of OC. Subsequently, the evaluation of CDCA5 expression was conducted in 10 samples taken from epithelial OC tissues and an equivalent number of normal ovarian tissues (Table S1). The Western blot (WB) analysis confirmed the upregulated expression of CDCA5 protein in the OC tissues compared to the normal tissues (*p* < 0.05, Fig. 1D, E). The expression of CDCA5 mRNA was also found to be significantly upregulated in the OC tissues in comparison to the normal tissues (Fig. 1F). Same results were obtained in the OC cell lines as well. The protein and mRNA levels of CDCA5 wereshown to be raised in human OC cell lines in comparison to ovarian epithelial cell lines. Among the cell lines tested, SKOV3 cells showed the highest expression, and the HEY and A2780 cells exhibitedthe lower expression (all *p* < 0.05, Fig. 1G,H, and I).

### CDCA5 promotes the proliferation of ovarian cancer cells

**Fig. 2:**
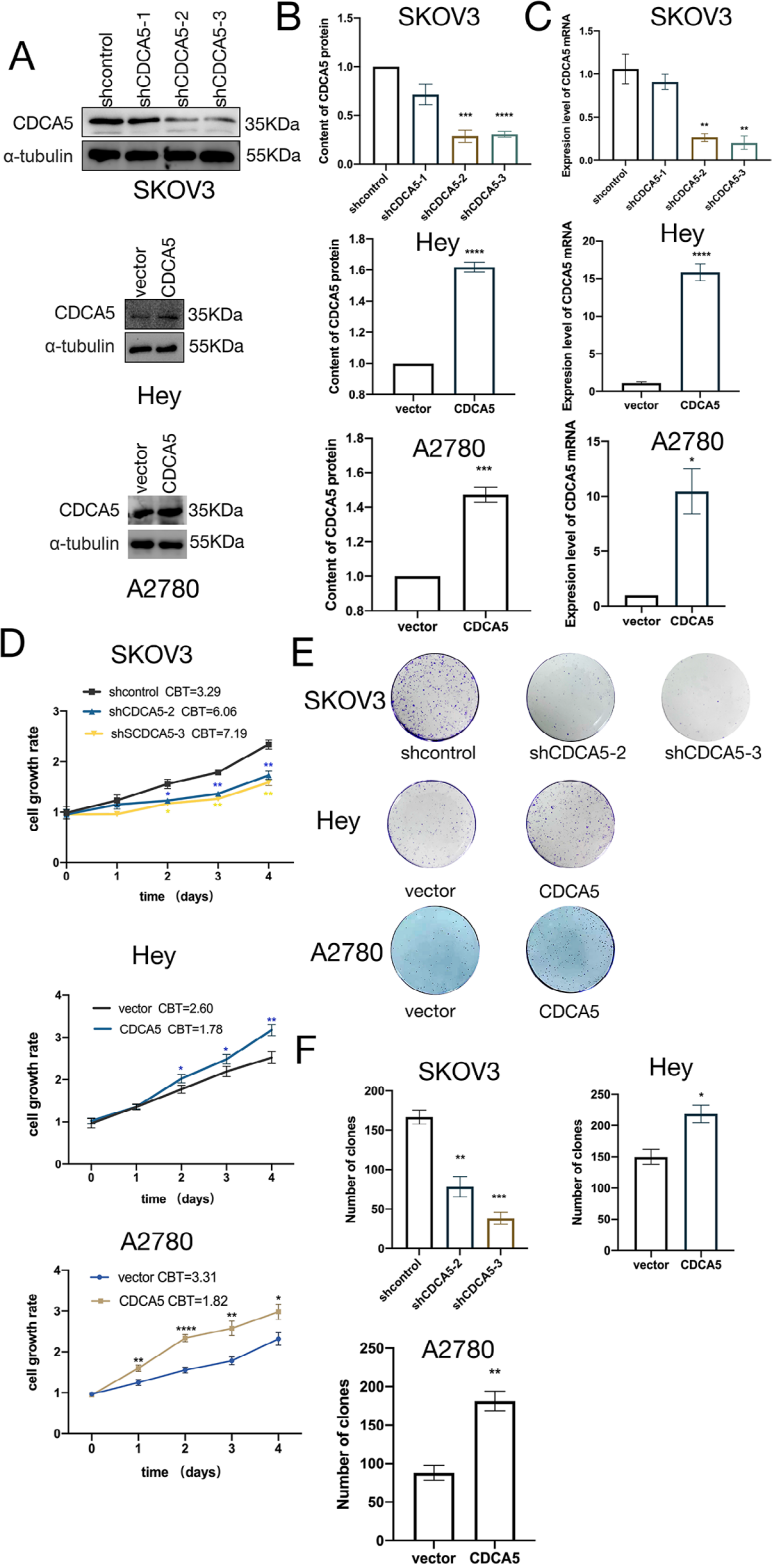
CDCA5 promotes proliferation in OC in vitro. **A,B** WB experimental analysis of CDCA5 expression in SKOV3 cell line by stable expression of shCDCA5 (shCDCA5-1, 2, and 3) or control vector and in HEY and A2780 cells stable expression of empty vector or CDCA5. **C** Determination of CDCA5 mRNA levels SKOV3,HEY and A2780 cells. **D** CCK-8 assay analyzed the cellular viability of SKOV3/sh2, sh3 and SKOV3/NC, HEY/CDCA5, A2780/CDCA5, and control vector. **E,F** Colony forming assay was conducted in SKOV3,HEY and A2780

To study the role of CDCA5 in OC, the CDCA5-shRNA lentivirus was utilized to infect the OC cell lines with high CDCA5 expression. The SKOV3 cell line was subjected to transfection with shCDCA5-1, shCDCA5-2, and shCDCA5-3. The effectiveness of knockdown in the shCDCA5-2 and shCDCA5-3 groups was confirmed using RT-qPCR and WB analysis. The HEY and A2780 cells were subjected to stable transfection with a gene vector expressing human CDCA5. The transfection process was effective, as confirmed by the subsequent overexpression of CDCA5 (all *p* < 0.01, Fig. 2A, B, and C). The results of the CCK8 experiments demonstrated a substantial reduction in the viability of SKOV3 cells in vitro following the CDCA5 knockdown. On the other hand, the ectopic overexpression of CDCA5 resulted in a notable increase in the proliferation of HEY and A2780 cells (Fig. 2D). The colony-forming test provided additional support for this interpretation (Fig. 2E and F).

### CDCA5 promotes the proliferationand migration but inhibits the dna damage and apoptosis in ovarian cancer cells

**Fig. 3:**
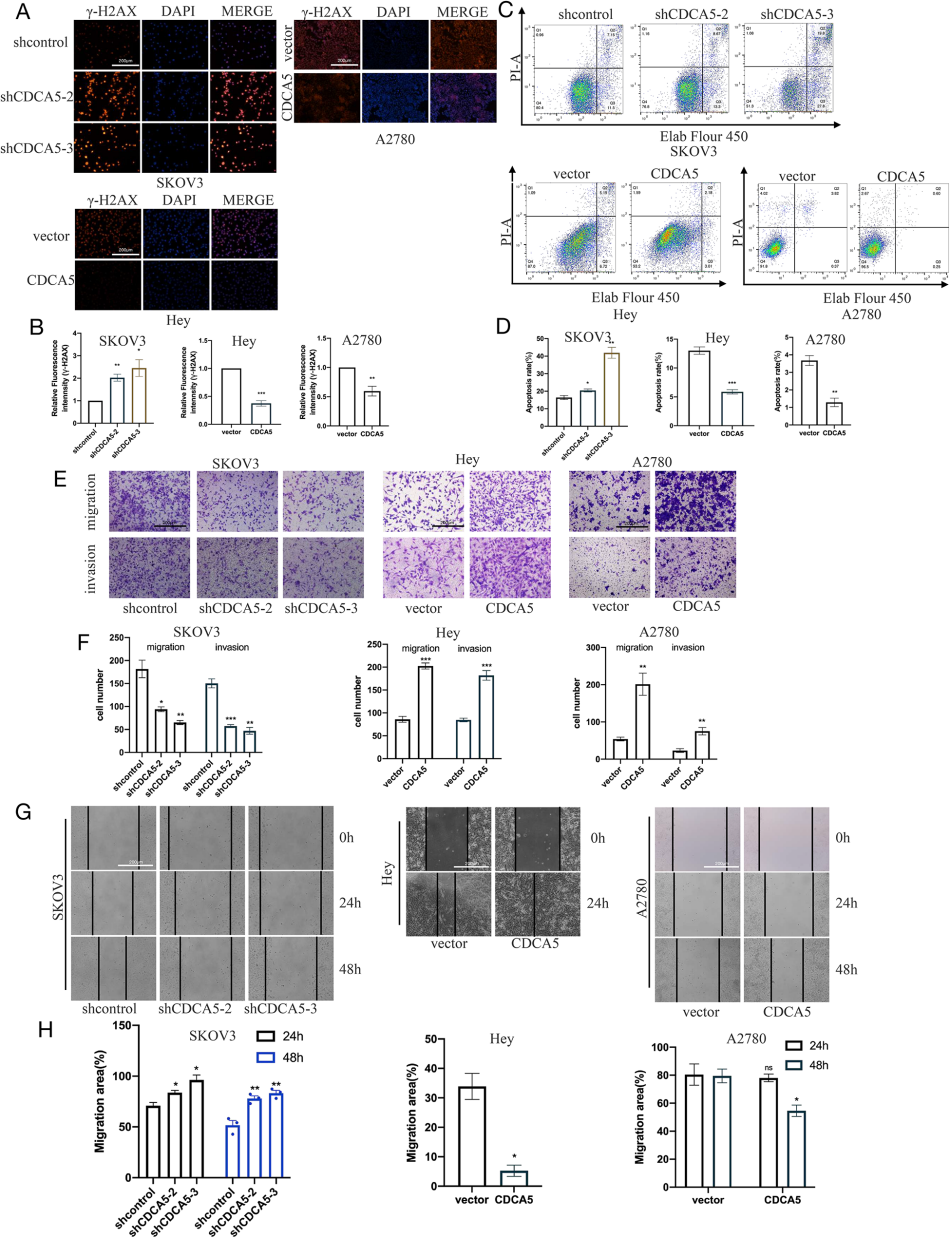
CDCA5 inhibits DNA damage and apoptosis but promotes invasion and migration in OC in vitro. **A,B** Immunofluorescence assay assessed the DNA damage and revealed a percentage of γ-H2AX-positive signals following overexpression or knockdown of CDCA5 in SKOV3,HEY and A2780 cells compared to the control cells. **C,D** Evaluation of apoptosis in SKOV3,HEY and A2780 cells by Flow cytometry. **E,F** Cellular invasion and migration in SKOV3,HEY and A2780 cells by performing transwell assay. **G,H** In SKOV3,HEY and A2780 cells, a wound healing assay was performed

Detection of γ-H2AX by immunofluorescence revealed that γ-H2AX expression was higher at CDCA5 inhibition in SKOV3 cells, representing increased DNA damage. In contrast, the immunofluorescence labeling analysis demonstrated a reduction in the development of γ-H2AX foci (red) following the overexpression of CDCA5 in HEY and A2780 cells (Fig. 3A and B). The results obtained from flow cytometry analysis demonstrated a significant increase in apoptotic cells in the shCDCA5 groups in comparison to the normal control groups. Conversely, the overexpression of CDCA5 led to a notable decrease in cell apoptosis (Fig. 3C and D). The results obtained from the woundhealing (Fig. 3E and F) and the transwell (Fig. 3G and H) experiments demonstrated that the expression of knockdown CDCA5 resulted in a decrease in the migratory and invasive capabilities of the cells. Conversely, the overexpression of CDCA5 was shown to improve cell migration and invasion.

### Knockdown of CDCA5 inhibited tumorigenicity of ovarian cancer cells in vivo

**Fig. 4:**
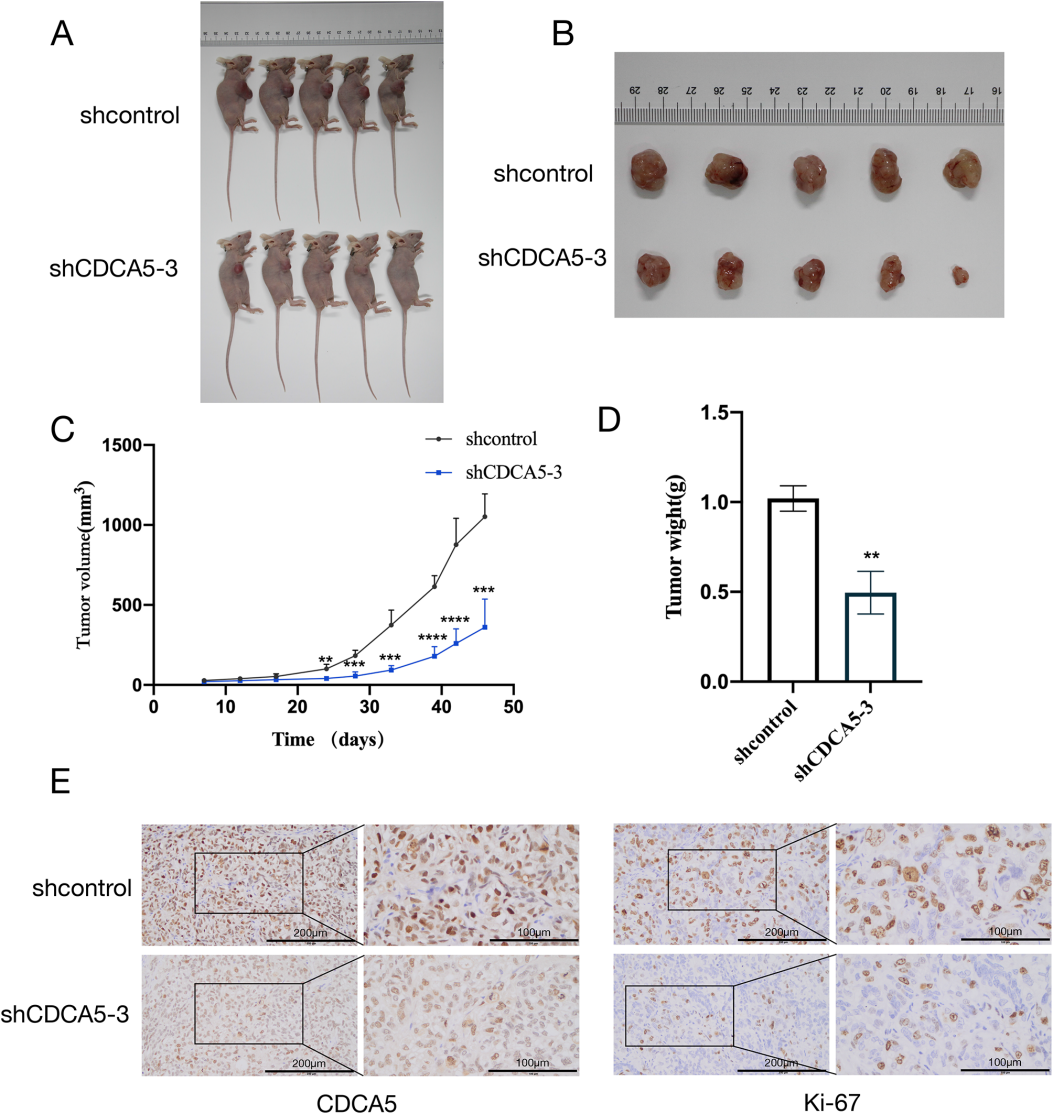
Knockdown of CDCA5 suppresses tumorigenesis and tumor growth of SKOV3 cells in vivo. **A** Images of miceOC tumors inoculated with SKOV3/NC and SKOV3/sh3 cells. **B** Tumor photographs were shown. **C** The tumor growth curves of mice after tumor inoculation. **D** Tumors weight of each group. **E** IHC staining image of Ki-67 and CDCA5 in OC tumor tissues (left:200 x, right:400 x)

In order to assess the role of CDCA5 in the growth and progression of OC tumors in vivo, the SKOV3 cell line underwent stable transfection using a lentivirus system with either an NC or shCDCA5-3. Subcutaneous injection of cells in the left axillary areas of nude mice was performed to monitor tumor growth for 7 to 50 days post-implantation beforetheir removal for examination. Knockdown of CDCA5 significantly reduced the tumor volume (Fig. 4A and B). The group treated with shCDCA5 exhibited significant inhibition of tumor growth curves and weights compared to the NC (*p* < 0.01, Fig. 4C and D). Ultimately, each relevant markerwas validated by immunohistochemistry (IHC),demonstrating that the shCDCA5 group’s CDCA5 and Ki67 expression levels were much lower than those of the NC group (Fig. 4E).

### TGF-β1/smads pathway is required for CDCA5 to promote invasive migrationin ovarian cancer

**Fig. 5:**
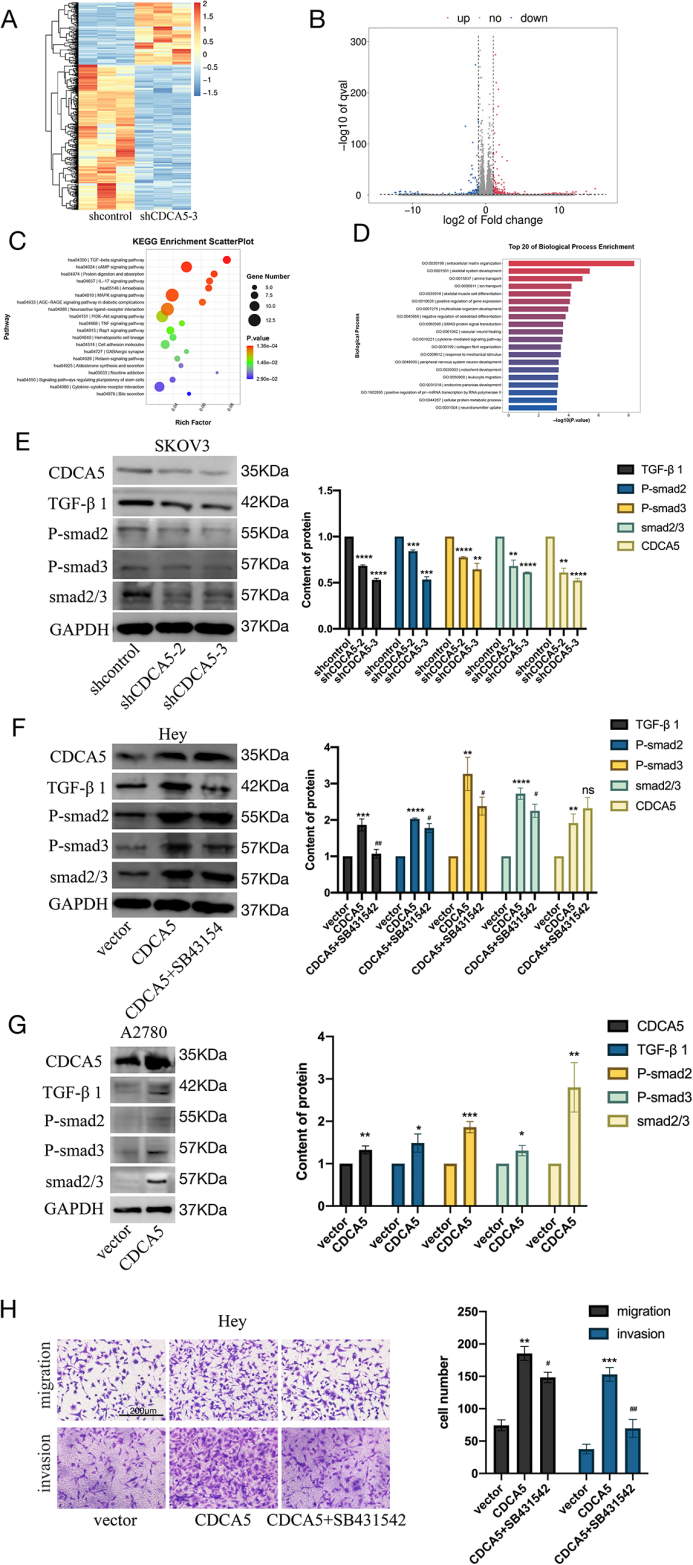
TGF-β1/smads pathway is essential for CDCA5-mediated invasion and migration in OC. **A** Heatmap showing differentially expressed genes among SKOV3/NC and SKOV3/sh3 cells. **B** Volcano plot shows differentially expressed genes between SKOV3/NC and SKOV3/sh3 cells. **C** The biological process with the top 20 enrichments is shown. **D** The top 20 functionally enriched KEGG pathways were shown in a scatter plot. **E** The signal transduction proteins in SKOV3 cells were detected by WB. **F** WB analysis explored the expression of TGF-β1,smad2/3, p-smad2, and p-smad3 with SB431542 (TGF-β1 inhibitor, 10 µM) in HEY cells. **G** The signal transduction proteins in A2780 cells were detected by WB. **H** Transwell assays were performed to investigate the invasion and migration of CDCA5 in HEY cells treated with or without SB431542. (*means CDCA5 group compared with vector, #means CDCA5 group compared with or without SB431542)

To investigate the probable pathway of CDCA5 in OC, the lentivirus system was used to transfect SKOV3 cells with either an NC or shCDCA5-3. Subsequently, RNA-seq was performed to analyze the alterations between the two experimental groups. The volcano plot and heatmap were used to visualizethe genes that exhibited differential expression between shCDCA5-3 and control SKOV3 cells (Fig. 5A and B). The Gene Ontology (GO) analysis revealed a significant enrichment of biological processes related to the organization of the ECM (Fig. 5C). Kyoto Encyclopedia of Genes and Genomes (KEGG) analysis revealed that silencing CDCA5 substantially affected the TGF-β pathway, suggesting a critical impact of TGF-β pathway in the pro-invasiveness of CDCA5 in OC (Fig. 5D).

The WB analysis showed that the downregulation of CDCA5 in SKOV3 cells significantly decreased the expression levels of TGF-β1, Smad2/3, p-Smad2, and p-Smad3 (Fig. 5E). In HEY and A2780 cells, overexpression of CDCA5 significantly upregulated TGF-β1, Smad2/3, p-Smad2, and p-Smad3 expression (Fig. 5F and G). Moreover, after treating OE-CDCA5 HEY cells with the TGF-1/Smads pathway inhibitor SB431542, a decrease in the expression of TGF-β1, Smad2/3, p-Smad2, and p-Smad3 was observed (Fig. 5F). In addition, OE-CDCA5 HEY cells treated with SB431542 partially reversed their invasion and migration capacities in the transwell (Fig. 5H). Hence, the upregulation of CDCA5 activated the TGF-β1/Smad2/3 pathway, facilitating the migration and invasion of OC cells.

## Discussion

The improvement of PFS in patients with OC has not made significant strides in recent decades, althoughdisease-specific survival has been prolonged [15]. Hence, identifying the key determinants responsible for advancing OC continues to pose a significant therapeutic obstacle. The present work revealed a considerable upregulation of CDCA5 in both OC tissues and cell lines and also showed that CDCA5 facilitated the growth of OC. The current study presented that the oncogenic characteristics of CDCA5 in OC cells are linked to the activation of a transcriptional program brought on by the downgrading of CDCA5. This transcriptional pathway promotes the activation of biological processes involved in metastasis, including leukocyte movement, ECM organization, and Smad protein signal transduction. Finally, it was shown that CDCA5 promoted motility and metastasis in OC cells by activating the TGF-β1 pathway. The present findings revealed that CDCA5 might serve as a viable therapeutic target for OC treatment.

Sororin, encoded by CDCA5,is one of the cell cycle-dependent mediators essential for sister chromatid cohesion during mitosis [16]. Later, it was found that sororin knockdown caused mitotic arrest in cancer cells [17]. Due to recent advancements in bioinformatics, it is now known that CDCA5 is linked to poor prognosis of various carcinomas [18]. The current study also utilized bioinformatics tools and found that CDCA5 facilitated cell proliferation, affected DNA damage, and impeded apoptosis in the OC. The γ-H2AX was detected using an immunofluorescence assay, which indicated damage to the DNA. The CDCA5-silenced OC cells showed increased DNA damage compared to the control cells. Another study done on renal cancer cells showed similar results [19]. In light of prior research, it can be postulated that CDCA5 might protect DNA from harmful damage by controlling chromosomal stability. Notably, the DNA damage response mechanisms in OC are potential therapeutic targets. The effective development of poly-ADP-ribose polymerase inhibitors (PARPi) has resulted in a novel approach to treating this condition. As a result, the impact of CDCA5 on DNA damage response may serve as the starting point for future OC treatments.

There was no significant difference observed in the protein expression levels of CDCA5 in the cancer tissues of the 10 cases included in this study, despite comprising three partial response cases and seven complete response cases. Considering the limited sample size, it is insufficient to thoroughly investigate the relationship between CDCA5 and treatment response. In our cell experiments, we not only utilized SKOV3 and HEY cells from ovarian serous adenocarcinoma but also incorporated A2780 cells from ovarian endometrioid adenocarcinoma. It was found that CDCA5 plays a consistent pro-oncogenic role across different subtypes of ovarian cancer cell lines. Moreover, it is worth noting that Hey cells are p53 wild-type while SKOV-3 cells are p53 mutant-type. Interestingly, our study revealed higher expression of CDCA5 in p53 mutant-type SKOV3 cells, which aligns with previous biosignature studies indicating a correlation between CDCA5 and the P53 pathway [20]. Therefore, further investigation into specific correlations between CDCA5 and P53 mutations is warranted for comprehensive understanding.

The analysis of transcriptomic alterations resulting from the knockdown of CDCA5 indicated that the biological process most prominently affected was the organization of the ECM. The molecular function encompasses the ECM structural components that impart tensile strength, and tumors frequently utilize ECM and remodel it to establish a microenvironment that facilitates its development and spread [21]. The tensile strength of the ECM promotes tumor invasion [22]. On the other hand, collagen contained in the ECM in the biological function of CDCA5 can also facilitate tumors’ ability to migrate [23]. The CDCA5 protein enhanced migration and invasion in cellular investigations, aligning with the findings from the sequencing experiments in a study showing the metastatic nature of breast cancer [11].

Furthermore, a correlation has been shown between ECM and the immune system [24]. The presence of CDCA5 has been found to exhibit a notable correlation with the infiltration of immune cells across a multitude of cancers within The Cancer Genome Atlas Program (TCGA) database. Furthermore, patients displaying elevated levels of CDCA5 expression have shown a more favorable response to anti-PD-L1 treatment [25]. The influence of CDCA5 in developing the immune microenvironment in OC has not been explored extensively.

In the early phases of cancer, the TGF-β showstumor-suppressive properties, whereas, in later stages, it promotestumor growth [26]. In the context of OC, TGF-β exerts tumor-promoting effects by suppressing the tumor microenvironment [27]. The role of TGF-βin the induction of EMT has been extensively studied, with evidence suggesting that it exerts its effects through activating the Smad family of transcription factors [28]. TGF-β1 has been reported to promote peritoneal metastasis of OC through a Smad-dependent pathway [29]. Currently, no research has been conducted to examine the association between CDCA5 and the TGF-β pathway. Previous research has mainly concentrated on investigating the AKT [11, 13] and ERK [30] signaling pathways. The KEGG enrichment analysis showed that the TGF-β signaling mechanism had the highest level of statistical significance in correlation. The TGF-β signaling pathway exhibits positive regulation by CDCA5. The functional role of CDCA5 in facilitating metastasis in OC cells was compromised when the TGF-β pathway was inhibited. Additionally, it was shown that the depletion of CDCA5 resultedin a downregulation of the phosphorylation of Smad2/3. A study observed that the oncogenic effects of TGF-β can be mediated by Smad2/3 even when Smad4 is absent [31]. Unfortunately, the current study failed to elucidate the specific molecular mechanism, which will be the direction of thefuture research endeavors. The aforementioned findings align with the transcriptome analysis that explains the oncogenic mechanism of CDCA5 in OC.

## Conclusions

In a nutshell, this study revealed a notable upregulation of CDCA5 expression in OC tissues and cell lines. Furthermore, the present findings show that CDCA5 is vital in facilitating the metastasis and proliferation of OC. The primary biological function of CDCA5 in OC cells was mainly centered on the ECM. Subsequent investigations revealed that CDCA5 was able to enhance the expression of genes located downstream of the TGF-β pathway, hence exerting an aiding influence on metastasis through activating the TGF-β1 pathway. This study provides novel insights into potential targets for therapeutic intervention in the treatment of OC.

## Methods

### Bioinformatics analysis

To compare the levels of CDCA5 mRNA expression in normal and OC tissues, a box plot was constructed using the GEPIA database(http://gepia.cancer-pku.cn). Log2 (TPM + 1) transformation was done to analyze the data of gene expression. The Kaplan-Meier(KM) plotter database (https://kmplot.com/analysis/) was utilized for the survival analysis. The patient cohort was stratified into two groups according to the median expression of CDCA5 (224753_at) in the KM Plotter for OC. Subsequently, the progression-free survival (PFS) and overall survival (OS) of these two groups were assessed and compared individually. The hazard ratio, 95% confidence interval, and logrank *p*-value are displayed adjacent to the curve. The number of patients in the group is indicated below the curve.

### Tissues and cell lines

A random sample of individuals was selected, from whom fresh samples of both normal ovarian tissue and OC tissue were obtained. There were ten cases for each kind of tissue. The aforementioned samples were obtained from the First Affiliated Hospital of Bengbu Medical University. The present study received approval from the Ethics Committee of Bengbu Medical University (Grant [2023] 390.). All patients signed the informed consent. Human OC cell lineswere purchased from Pricella Life Sciences Co. (Wuhan,China). Patient informations are displayed in Table S1. The A2780,HO8910, and OVCAR3 cells were cultured in RPMI-1640, whereas IOSE80, ES-2, and HEY cells in DMEM and SKOV3 cells in McCoy’s 5 A media. The cell lines were identified and confirmed using the short tandem repeat (STR) profiling. The information about cells is displayed in Table S2. The TGF-β pathway inhibitor, LY2157299, was acquired from Selleck Chemicals(USA).

### Quantitative reverse transcription PCR (qRT-PCR)

The total RNA extracted from the samples was performed using TRIzol (Biosharp, China). To synthesize cDNA from the isolated RNA, PrimeScript™ RT reagent Kit (Takara,China) was utilized. The VeriFiler™ Plus PCR (Thermo Fisher, USA) was used to perform the real-time PCR reaction. The primer pairs used in this study are displayed in Table S3.

### Western blot (WB)

Using a radioimmunoprecipitation (RIPA, Beyotime, China) technique, the total protein from the samples was extracted. The protein content was quantified using the bicinchoninic acid (BCA) protein assay kit. The protein samples were separated using a 12% SDS-PAGE gel and transferred onto a poly(vinylidene) difluoride (PVDF) membrane (Millipore, USA). The PVDF membrane was blocked by treating it with a 5% solution of skimmed milk, followed by a 2-hour incubation. The primary antibody was added, and the protein samples were incubatedat 4ºC overnight. Afterward, incubation of the sample with the secondary antibody was doneat room temperature (RT) for a duration of 1 h. Finally, for the visualization of the protein samples, the electrochemiluminescence (ECL) chromogenic solution (Beyotime, China) was utilized. The antibodies used in the study are documented in the Table S4.

### Lentivirus infection

SKOV3 cells were stably transfected with lentivirus vectors containing CDCA5 knockdown constructs (sh1, sh2, sh3) and vector control (NC) provided by Obio, Shanghai, China. The lentivirus system containeda puromycin selection marker. In addition, the overexpressed CDCA5 constructs were also stably transfected in the HEY cells using the lentivirus system (Obio, Shanghai, China), following the directions provided by the manufacturer. The sequences of the CDCA5 interference aredocumented in Table S5. Puromycin was used to select stable cells containing gene constructs. The cells were exposed to puromycin for two weeks at a dose of 1 µg/mL.

### Cell counting kit-8 (CCK-8) assay

For this experiment, a total of 2 × 10^3^ cells were evenly distributed into designated wells of a 96-well plate. Following apposition, the cells were replenished with a growth medium containing a 10% solution of CCK-8 and incubated for 1 h. The cellular supernatant was collected, and absorbance was measured at 450 nm wave length. The recorded data at this specific time point represents the initial measurement of cell growth, referred to as the 0-hour data. The subsequent measurements were taken at 24, 48, 72, and 96 h.

### Colony formation assay

In a 6-well plate, cells were seeded (5 × 10^2^per well) to assess colony formation. After 15 days of incubation, the culture plates were subjected to staining with crystal violet, and then images were captured.

### Immunofluorescence assay

For the immunofluorescence experiment, cells were seeded on a 24-well plate with a seeding density of 5 × 10^4^ cells per well. The climbing slices were placed, and 4% paraformaldehyde was used to fix the cells for 15 min following cell apposition. Afterwards, the cells were treated with an immunofluorescence permeation solution (Biyuntian) for 5 min. Blocking was performed by incubating the cellswith 10% goat serum for 1 h. Moreover, the primary antibody was prepared by mixing H2AX antibody (Abcam) at a dilution of 1:200 in 10% goat serum. The cells were kept with primary antibody at 4°C overnight. The secondary antibody, goat anti-rabbit IgG H&L (Alexa Fluor® 647, ab150079, Abcam), was then added and incubated for 2 h. The slices were blocked using a blocking solution containing DAPI (Beyotime) and subsequently photographed.

### Flow cytometry assay

Using a 6-well plate (2 × 10^5^cells/well), the flow cytometry assay was performed. The cells were centrifugated and resuspended in 500 µL of diluted annexin V binding buffer(Pricella, Wuhan, China). The staining solution (5 µL) was added and incubated for 15 min at RT in the dark to avoid contact with light. The flow cytometry investigation was conducted using a flow cytometer from BD Biosciences.

### Wound healing assay

In the wound healing assay, a 6-well plate seeded with cells (5 × 10^5^cells/well) was used. After the establishment of a monolayer, a linear mark was made on the cell monolayer to create a scratch (wound) in the monolayer. The serum-free medium was added, and images were taken. The time points for image acquisition were 0, 24, and 48 h after scratch induction.

### Transwell assays

For the transwell invasion experiment,the seeding density of cells in a serum-free medium was adjusted to 1 × 10^5^ cells/mL. A volume of 100 µL of stromal gel, diluted at a ratio of 1:10, was introduced into the upper chamber of the transwell. The upper chamber was also filled with cell suspension (100 µL), whereas the lower chamber contained a 500 µL culture medium with serum (10%). Incubation was done for 24 h, and the chambers were carefully removed. Subsequently, the samples were fixed using a 4% paraformaldehyde solution for 15 min. Following fixation, crystal violet was used for staining. The cells were observed using a microscope, and images were taken. The aforementioned procedures were repeated, excluding the stromal gel to simulate migratory conditions.

### In vivo xenograft tumor model

Female BALB/c nude rats (4–5 weeks) were procured from the Hangzhou Ziyuan Animal Technology Co laboratory. The animals being studied were housed in accordance with the standard conditions at the animal center of the First Affiliated Hospital of Bengbu Medical University. The mice were split into two groups via random selection, with each group consisting of 5 mice. Subcutaneous injections of 1 × 10^7^ SKOC3-NC cells and SKOV3-sh CDCA5 cells were administered to both groups of BALB/c nude mice in the right dorsal neck area. The tumor volume was monitored over two months, with measurements taken every 4–5 days using vernier calipers. The calculation of tumor volume was determined using the formula (width^2^ × length)/2 (mm^3^). The mice were euthanized using CO^2^inhalation asphyxiation, and the weight of the tumors was recorded. For IHCanalysis, tumor samples were isolated. The study protocol received approval from the Animal Ethics Clerks’ Council.

### Immunohistochemistry (IHC) analysis

Fixation was done with 4% paraformaldehyde, and paraffin was used for tissue embedding. The parafinized tissues were sliced into thin Sect. (4 μm thickness). The sections were then placed in a 60ºC oven for 2 h. After dewaxing and rehydration, antigen retrieval was performed using the sodiumcitrate antigen repair solution. The sections were blocked by incubating them with 10% goat serum for 30 min. Thesections were incubated with the primary antibody at 4ºC overnight. Afterwards, the secondary antibody (1:100) was added at RT for 30 min. The DAB (diaminobenzidine) color development solution was applied for 5 min, after which the sample was rinsed with distilled water. Hematoxylin was used to stain the nuclei, followed by a process of dehydration and subsequent sealing with a transparent film to facilitate microscopic inspection. The primary antibodies employed in the investigation are recorded in Table S6.

### RNA sequencing and bioinformatics analysis

The high-throughput mRNA-Seq assay was performed using Lianchuan Biomarker Technologies(Hangzhou, China). Briefly, RNA was purified from the SKOV3-sh3 and SKOV3-NC cells using TRIzol reagent and Illumina Novaseq™ 6000 (LC Bio-Technology CO., Ltd. Hangzhou, China) was used for sequencing. Gene expression was quantified using Fragments Per Kilobase of transcript per Million mapped reads (FPKM), and the analysis of differential genes between the samples was conducted using the edgeR package with a difference multiple > 2-fold or < 0.5-fold,and *p* < 0.05 was considered statistically significant. Using the Database for Annotation, Visualization, and Integrated Discovery (DAVID) tool, the genes were submitted to enrichment analysis for the GO and KEGG.

### Statistical analysis

All experimental data are represented as mean ± standard error of the mean (mean ± SEM). The statistical analysis conducted involved one-way ANOVA and student’s t-test. The *p* < 0.05 was statistically significant. The statistical significance is given as * *p* < 0.05, **p < 0.01, ***p < 0.001, and **** *p* < 0.0001.

### Electronic supplementary material

Below is the link to the electronic supplementary material.

Supplementary Material 1

Supplementary Material 2

Supplementary Material 3

Supplementary Material 4

Supplementary Material 5

Supplementary Material 6

## Data Availability

The datasets generated or analysed during the current study are available from the corresponding author on reasonable request.

## Abbreviations

CDCA5: cell division cycle associated 5
ECM: extracellular matrix
EMT: epithelial-mesenchymal transition
OC: ovarian cancer
TGF: transforming growth factor
WB: western blot
