# Supplementary material for: CDCA5 promoted cell invasion and migration by activating TGF-β1 pathway in human ovarian cancer cells
Source: J Ovarian Res. 2024 Mar 27;17:68. doi: 10.1186/s13048-024-01393-5 (PMC10967103; doi:10.1186/s13048-024-01393-5)
Supplement: Supplementary file 1 — Supplementary Material 1 [file 13048_2024_1393_MOESM1_ESM.docx]

| Tissue No. | Ages | Stages（FIGO） | Histological subtypes | Treatment responses |
| --- | --- | --- | --- | --- |
| N1 | 52 | - | Normal ovarian tissue | - |
| T1 | 69 | Ⅲc | High grade serous carcinoma | CR |
| N2 | 53 | - | Normal ovarian tissue | - |
| T2 | 46 | Ⅲc | High grade serous carcinoma | CR |
| N3 | 55 | - | Normal ovarian tissue | - |
| T3 | 57 | Ⅳa | High grade serous carcinoma | PR |
| N4 | 52 | - | Normal ovarian tissue | - |
| T4 | 57 | Ⅲc | High grade serous carcinoma | CR |
| N5 | 49 | - | Normal ovarian tissue | - |
| T5 | 59 | Ⅲc | High grade serous carcinoma | CR |
| N6 | 52 | - | Normal ovarian tissue | - |
| T6 | 50 | Ⅲb | High grade serous carcinoma | CR |
| N7 | 48 | - | Normal ovarian tissue | - |
| T7 | 58 | Ⅲc | High grade serous carcinoma | PR |
| N8 | 49 | - | Normal ovarian tissue | - |
| T8 | 60 | Ⅲc | High grade serous carcinoma | PR |
| N9 | 48 | - | Normal ovarian tissue | - |
| T9 | 71 | Ⅲc | High grade serous carcinoma | CR |
| N10 | 50 | - | Normal ovarian tissue | - |
| T10 | 65 | Ⅲc | High grade serous carcinoma | CR |

The demographics of the patients

Complete response (CR): disappearance of all tumour target lesions, no new lesions, and normal tumour markers for at least 4 weeks. Partial response (PR): ≥30% reduction in the sum of the largest diameters of the target lesions of the tumour, maintained for at least 4 weeks.
