# Supplementary material for: CDCA5 promoted cell invasion and migration by activating TGF-β1 pathway in human ovarian cancer cells
Source: J Ovarian Res. 2024 Mar 27;17:68. doi: 10.1186/s13048-024-01393-5 (PMC10967103; doi:10.1186/s13048-024-01393-5)
Supplement: Supplementary file 2 — Supplementary Material 2 [file 13048_2024_1393_MOESM2_ESM.docx]

| Cell line name | Histological subtypes | Treatment response | Mutation status |
| --- | --- | --- | --- |
| SKOV3 | Ovarian serous cystadenocarcinoma | Certain degree of resistance to diphtheria toxin, cisplatin and adriamycin | APC,  FBXW7,PIK3CA,TP53 |
| HEY | High grade ovarian serous adenocarcinoma | Certain degree of resistance to cisplatin | BRAF,  KRAS |
| OVCAR3 | High grade ovarian serous adenocarcinoma | Certain degree of resistance to adriamycin, cis-chloroplatin and levophenylalanine nitrogen mustard (melphalan) | PIK3R1,TP53 |
| A2780 | Ovarian endometrioid adenocarcinoma | No exposure to any anti-cancer drugs or chemicals | ATM,  PTEN |
| ES-2 | Ovarian clear cell adenocarcinoma | Low to moderate tolerance to a wide range of chemotherapeutic agents including adriamycin, cisplatin, cacodyl mustard, etoposide; | BRAF,  PALB2,TERT,  TP53 |
| HO-8910 | Ovarian serous cystadenocarcinoma | - |  |
| IOSE-80 | Ovarian surface epithelial cell | - | - |

Information about the cell lines
