# Supplementary material for: CDCA5 promoted cell invasion and migration by activating TGF-β1 pathway in human ovarian cancer cells
Source: J Ovarian Res. 2024 Mar 27;17:68. doi: 10.1186/s13048-024-01393-5 (PMC10967103; doi:10.1186/s13048-024-01393-5)
Supplement: Supplementary file 3 — Supplementary Material 3 [file 13048_2024_1393_MOESM3_ESM.docx]

**Table S3** Sequences of Primer for qRT-PCR.

| **CDCA5** |  |
| --- | --- |
| Forward | 5’-GACGCCAGAGACTTGGAAATG-3’ |
| Reverse | 5’- GGACCTCGGTGAGTTTGGAG-3’ |
| **GAPDH** |  |
| Forward | 5’- ACCACAGTCCATGCCATCAC-3’ |
| Reverse | 5’-TCCACCACCCTGTTGCTGTA -3’ |
