# Supplementary material for: CDCA5 promoted cell invasion and migration by activating TGF-β1 pathway in human ovarian cancer cells
Source: J Ovarian Res. 2024 Mar 27;17:68. doi: 10.1186/s13048-024-01393-5 (PMC10967103; doi:10.1186/s13048-024-01393-5)
Supplement: Supplementary file 4 — Supplementary Material 4 [file 13048_2024_1393_MOESM4_ESM.docx]

**Table S4**Antibodies were used to WB.

| **Antibodies** | | |
| --- | --- | --- |
| **REAGENT** | **Product No. and Source** | **Dilution** |
| CDCA5 | ab240328, Abcam | 1:1000 |
| TGF beta 1 Antibody | AF1027, Affinity | 1:1000 |
| Smad2/3 Antibody | AF6367, Affinity | 1:1000 |
| Phospho-Smad2 (Ser250) Antibody | AF3450, Affinity | 1:1000 |
| Phospho-Smad3 (Ser425) Antibody | AF3362, Affinity | 1:1000 |
| Tubulin | AF1216,Beyotime | 1:5000 |
| GAPDH | 60004-1-lg,Proteintech | 1:5000 |
