# Supplementary material for: CDCA5 promoted cell invasion and migration by activating TGF-β1 pathway in human ovarian cancer cells
Source: J Ovarian Res. 2024 Mar 27;17:68. doi: 10.1186/s13048-024-01393-5 (PMC10967103; doi:10.1186/s13048-024-01393-5)
Supplement: Supplementary file 5 — Supplementary Material 5 [file 13048_2024_1393_MOESM5_ESM.docx]

**Table S****5**TargetSeq of the interference CDCA5 are the followings.

| **shCDCA5 1** |
| --- |
| CGAGAAACAGAAACGTAAGAA |
| **shCDCA5 2** |
| GAAGCTGCTGAGCAGTTTGAT |
| **shCDCA5 3** |
| GCCAGAGACTTGGAAATGTCT |
