# Supplementary material for: CDCA5 promoted cell invasion and migration by activating TGF-β1 pathway in human ovarian cancer cells
Source: J Ovarian Res. 2024 Mar 27;17:68. doi: 10.1186/s13048-024-01393-5 (PMC10967103; doi:10.1186/s13048-024-01393-5)
Supplement: Supplementary file 6 — Supplementary Material 6 [file 13048_2024_1393_MOESM6_ESM.docx]

**Table S6** Antibodies were used to IHC.

| **Antibodies** | | |
| --- | --- | --- |
| **REAGENT** | **Product No. and Source** | **Dilution** |
| CDCA5 | ab240328, Abcam | 1:200 |
| Ki67 | MA5-14520, Invitrogen | 1:200 |
